# Ironbark: Developing a healthy community program for older Aboriginal people

**DOI:** 10.1002/hpja.581

**Published:** 2022-02-22

**Authors:** Rona Macniven, Aaron Simon, Roland Wilson, Adam Howie, Georgia Stewart, Tracey Ma, Norma Jean Turner, Sallie Cairnduff, Julieann Coombes

**Affiliations:** ^1^ School of Population Health UNSW Sydney Sydney New South Wales Australia; ^2^ Faculty of Health, Medicine and Human Sciences Macquarie University New South Wales Australia; ^3^ NSW Health Sydney New South Wales Australia; ^4^ 1065 Southgate Institute for Health, Society, and Equity Flinders University Adelaide South Australia Australia; ^5^ Ngarruwan Ngadju First Peoples Health and Wellbeing Research Centre, Australian Health Services Research Institute The University of Wollongong Wollongong New South Wales Australia; ^6^ 211065 The George Institute for Global Health Sydney New South Wales Australia

**Keywords:** Aboriginal and Torres Strait Islanders, ageing, community‐based intervention, older people, participatory action research

## Abstract

**Issue addressed:**

Programs by, with and for Aboriginal older people must be culturally safe and relevant. Successful elements include being Aboriginal specific and group based. Co‐design with Aboriginal people and stakeholders is essential. We describe the co‐design process of developing the *Ironbark: Healthy Community* program.

**Methods:**

Aboriginal ways of knowing, being and doing and yarning conversational methods guided the development process, during 2018. A desktop review provided details of current group characteristics and key community stakeholders. Stakeholder engagement regarding views about group operations, participants and benefits also occurred. Aboriginal Elders views of their groups were gathered through yarning circles in New South Wales (NSW). Grounded theory approach was used to ascertain key themes.

**Results:**

Initial engagement occurred with 13 different community stakeholders and organisations in three Australian states (NSW, South Australia (SA), Western Australia (WA)). Three yarning circles occurred with Elders from urban (N = 10), regional coastal (N = 10) and regional country (N = 4) groups. Six key themes were organised in three groups according to an Aboriginal ontology. 1. Knowing: groups provide opportunities to share knowledge and connect socially. Adequate program resourcing and sustainability are valued. 2. Being: groups strengthen culture, providing important social, emotional and other forms of support to age well. 3. Doing: previous program experiences inform perceptions for new program operations. Group venues and operational aspects should be culturally safe, acknowledging diversity among Elders, their preferences and community control. Themes were used to develop the program and its resource manual that were finalised with stakeholders, including steering committee approval.

**Conclusions:**

Stakeholder feedback at multiple stages and Aboriginal Elders’ perspectives resulted in a new co‐designed community program involving weekly yarning circles and social activities.

So what?: Co‐design, guided by Aboriginal ways of knowing, being and doing, can develop programs relevant for Aboriginal people.

## 1 INTRODUCTION

Aboriginal peoples are the world’s oldest continuous cultures. Aboriginal Elders are keepers of cultural knowledge, wisdom and are respected, trusted community leaders and educators, passing traditions to younger people and support maintaining connection to Country.^1^ These practices are an integral part of Aboriginal cultures. As Aboriginal people age, it is vital that they are enabled and supported to continue as cultural leaders, particularly as Aboriginal people have a lower life expectancy than other Australians, due to structural determinants.^2^ A previous study with 76 Aboriginal older adults found that healthy ageing was considered essential to continue to share knowledge of history and cultures, yet Aboriginal people may require greater support at a younger age.^3^

To be successful, programs by, with and for Aboriginal Elders must be culturally safe and relevant.^3^ Aboriginal‐specific, group‐based programs with continuity and flexibility have also been identified as important elements.^4^ Co‐design can be defined as meaningfully involving end‐users in research.^5^ Co‐design with Aboriginal people and community organisations as end‐users is essential when developing new programs and research.^6^ Implementing and evaluating initiatives delivered through Aboriginal community‐controlled organisations and by Aboriginal staff, and the involvement of these stakeholders and Aboriginal Elders in guiding co‐design processes is also necessary.^7^

A recently developed program and resource manual for Aboriginal Elders, *Ironbark: Standing Strong and Tall*, aims to prevent falls and was co‐designed with Aboriginal communities in New South Wales (NSW).^4^ The program was evaluated with six communities and 98 Aboriginal people aged 40‐90 years old; significant improvements in strength and balance and reductions in body mass index were achieved.^8^ There was also a noticeable increase in participants becoming involved with social activities and outings, including walking groups. All participants reported that they enjoyed the program and were willing to recommend it to others, with the yarning circles being the most popular program element.^8^

A cluster non‐randomised controlled trial is being conducted to test if the *Ironbark: Standing Strong and Tall program* prevents falls and improves health and well‐being among older Aboriginal people, compared to the *Ironbark: Healthy Community* program (www.ironbarkproject.org.au).^9^ The trial has strong Aboriginal leadership takes place in NSW as well as in South Australia (SA) and Western Australia (WA) where there are existing partnerships with senior Aboriginal researchers with relevant expertise and interest. Co‐design of the comparison program and resource manual is also important to ensure that it is relevant and accessible. A steering committee, comprises state level policy representatives and Aboriginal community‐controlled organisations, virtually meet quarterly and provide guidance and approval in all project aspects.

We describe the co‐design process of developing the *Ironbark: Healthy Community* program with community stakeholders and older Aboriginal people.

## 2 METHODS

Indigenous research methodology adopts Aboriginal ways of knowing, doing and being.^10^ Yarning circles are an Aboriginal research method for conversation that involves storytelling and knowledge sharing.^11^ Yarning has been used in Aboriginal communities for thousands of years and is central to building respect, learning from each other equally and preserving cultural knowledge and tradition.^11^

The two‐phased development process occurred during 2018, conducted by Aboriginal researchers in NSW. First, a desktop review was conducted to identify information about existing groups and services for Aboriginal Elders including their funding sources, focus, operational details and locations. Key community stakeholders and organisations in NSW, SA and WA involved with groups were identified through this review, and existing community relationships. Using purposeful sampling,^12^ stakeholders were invited to share their experiences in a semi‐structured telephone or face to face interview, depending on travel feasibility and were asked a series of six questions about their group operations, participants and perceived benefits (Table 1).

**TABLE 1:** Community and organisation stakeholder questions

1. Where does your Elders group get support from? (eg, through Aboriginal Community Controlled Health Services (ACCHSs), Land Council, other organisation)
2. How does your Elders group function (eg, how many times a week do you meet? What are the main things you do as a group?)
3. Who normally comes (eg, age range, men/women, how many people come)?
4. If you had some support, what would be the kinds of things you would like to do?
5. What are the main benefits of the Elders group for people who are part of the group?
6. What are the main benefits your community gets out of your Elders group?

Second, stakeholders with Elders groups were also asked if they would be interested in inviting their Elders to participate in yarning circles ref. Three groups of male and female Aboriginal Elders in geographically diverse regions of NSW were purposefully recruited to participate in yarning circles at each service, in line with local community protocols. These were conducted by an Aboriginal male project officer (AS) trained in yarning methods. The yarning groups used a semi‐structured format that asked what the Elders do as a group, how the Elders group runs, how the Elders groups benefits participants and your community and what activities or topics they would like to do in future Elders groups. The three yarning groups were an average of one hour in duration. The yarning circles were audio‐recorded and transcribed verbatim by a professional service. Data were coded and analysed by two Aboriginal and one non‐Aboriginal researchers using a grounded theory approach to group key themes within the three categories of Aboriginal ontology: knowing, doing and being.^10^

## 3 RESULTS

Engagement with 13 different community stakeholders and organisations during Phase 1 occurred between May and August 2018. These stakeholders and organisations included Aboriginal Community Controlled Health Services (ACCHSs), Aboriginal Land Councils, and the peak NSW, SA and WA Aboriginal health organisations.

Findings indicated stakeholder preferences were for activities that reflected the interests of Elders. These activities included art and outings although transport was noted as an issue. Elders of all ages above 40 years, with male and female participants in existing groups. Group size varied from 10 to 60 people. Some group venues were open for Elders to attend on all weekdays, or some groups met together on a particular weekday. Groups were considered important to communities and their host organisation and were often initiated by the community. Perceived benefits of the groups included keeping Elders active in the community, learning language, regular trips to a nearby larger town, providing support to, and by, Elders in the community. Support for groups was provided by government, land councils, donations and self‐funding.

**FIGURE 1:**
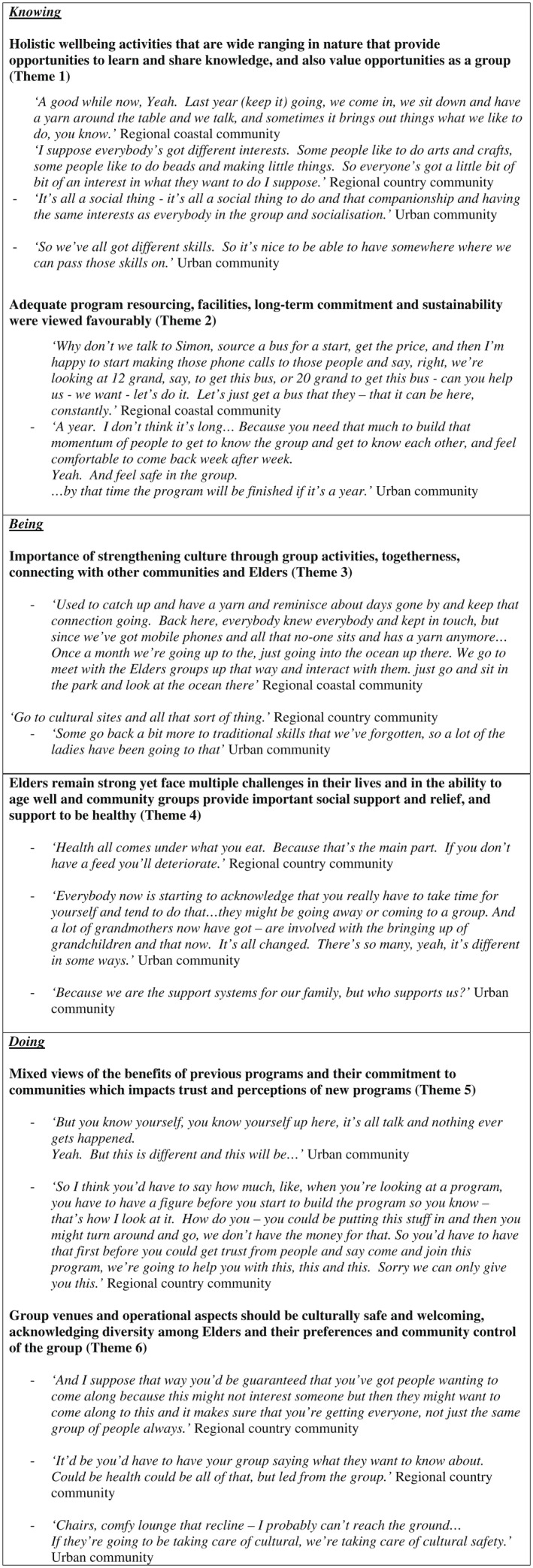
Yarning groups’ themes grouped within three categories of Aboriginal ontology: Knowing, doing and being

Three group yarning circles were conducted between July and September 2018. One group was in an urban area (N = 10); two groups were in regional areas, one coastal (N = 10) and one country (N = 4). Six key themes emerged from the yarning groups. These themes, grouped within three categories of knowing, doing and being,^10^ with supporting quotes are presented in Figure 1.

Findings from the stakeholder engagement, yarning circles and desktop review were incorporated to develop the *Ironbark: Healthy Community* program and resource manual. This process involved the team identifying activities preferred by Elders and their benefits in the yarning circle transcripts, using stakeholder consultations and the desktop review to categorise activities into different types. Categories included community led sessions, service provider workshops, outings, social activities and longer‐term projects. An outline of the program manual structure and categories was developed and refined by the (primarily Aboriginal) Ironbark team, in consultation with the project’s cultural advisor and steering committee. The manual design and layout were based on other Aboriginal resources and included artwork by an Aboriginal artist. Feedback on these draft materials were subsequently sought from participating services via email, but with no substantive changes suggested, with final approval given by the steering committee.

## 4 DISCUSSION

We report on the co‐design process of developing the *Ironbark: Healthy Community* program. The program aims to provide older Aboriginal people with opportunities to socialise, yarn and learn more about topics of relevance to them and their communities.^9^, ^13^ The weekly program involves a 1‐1.5‐hour yarning circle and/or other social activity. Participating sites will receive the manual, training, and support, including two paid positions to coordinate the group and deliver the program. The manual provides guidance on conducting yarning circles, ideas for different types of activities and example education sessions, whilst giving choice to the group. It is a living document that can be updated with feedback from implementation by groups, providing the opportunity for ongoing co‐design, reflecting experiences of Aboriginal Elders.

A recent study of the adaptation of an early childhood program for Aboriginal and Torres Strait Islander people noted the importance of community consultation and building relationships,^14^ that were also essential in the development of this program. Co‐design with Aboriginal communities is increasingly used in program and resource development.^6^ Similar approaches have recently engaged Aboriginal youth and an Aboriginal reference group to design a pre‐conception learning resource^15^ and Aboriginal people living with a disability to co‐design a toolbox of activities and associated program.^16^

Community collaboration and Aboriginal research methodological approaches are vital to ensure cultural safety and relevance.^17^ We used Indigenous research methods, specifically Aboriginal ontology of knowing, doing and being,^10^ and yarning with Aboriginal Elders to gain their perspectives of important program elements.^11^ These ontology concepts are both distinct and may overlap; this was evident in the six themes that arose from the yarning circles with Elders that were broadly consistent with stakeholder views. Beyond using these findings in program development, the yarning circle themes contribute to existing evidence about why Elders groups are important, their impact and contribution to communities.

We found that groups provide opportunities to share knowledge and connect socially. Elders valued adequate program resourcing, facilities and sustainability (Knowing). This is consistent with a recent review that highlighted that community engagement and sufficient resourcing are critical to implementation of programs by, with and for Aboriginal people.^18^ Elders expressed the importance of strengthening culture through the group that provides important social and other forms of support to live strong and age well (Being). Recent narratives from ageing Aboriginal women similarly demonstrated their ability to adapt to change, keeping identities and cultures strong.^19^ Elders shared their experiences of previous programs and how these inform their perceptions of how new programs should operate to be relevant and culturally safe (Doing). Culture enhances identity, well‐being and resilience.^20^ Four aspects of social networks, inclusiveness, empowerment, and connections were found to create a safe place to learn about cultures and Country, supporting an environment for strengthening identity, improving health and building resilience. These aspects resonate with these six themes, giving collective learnings of the benefits of Elders.

Strengths include Aboriginal leadership in all processes, yarning circles and community‐controlled stakeholder engagement directly informing the program and resource development. A limitation is a lack of opportunity to revisit the regional groups for feedback on the resulting program and manual, however ongoing informal discussions occurred with the urban group and other stakeholders.

## 5 CONCLUSION

The *Ironbark: Healthy Community* program was developed through a co‐design process that included stakeholder feedback at different stages and perspectives from Aboriginal Elders. Gaining insights from future experiences of Elders groups regarding program implementation and manual use will be valuable to learn about its impact and to inform future program planning.

## CONFLICT OF INTEREST

The authors declare no conflict of interest.

## ETHICS

Ethical approval was received from the Aboriginal Health and Medical Research Council (AH&MRC) Ethics Committee (140118) and noted by the University of New South Wales Human Research Ethics Committee. Written consent was obtained from all participants.

## Funding information

Australian National Health and Medical Research Council (NMHRC Project grant no. 1143085)
